# Structural Basis for ASPP2 Recognition by the Tumor Suppressor p73

**DOI:** 10.1016/j.jmb.2012.08.005

**Published:** 2012-11-02

**Authors:** Peter Canning, Frank von Delft, Alex N. Bullock

**Affiliations:** Structural Genomics Consortium, University of Oxford, Old Road Campus, Roosevelt Drive, Oxford OX3 7DQ, UK

**Keywords:** DBD, DNA-binding domain, OD, oligomerization domain, TEV, tobacco etch virus, TCEP, tris(2-carboxyethyl)phosphine, PDB, Protein Data Bank, TLS, translation/liberation/screw, TP73, 53BP2, transactivation, DNA damage, mutant p53

## Abstract

Tumor suppressors p53, p63 and p73 comprise a family of stress-responsive transcription factors with distinct functions in development and tumor suppression. Most human cancers lose p53 function, yet all three proteins are capable of inducing apoptosis or cellular senescence. Mechanisms are therefore under investigation to activate p73-dependent apoptosis in p53-deficient cancer cells. Significantly, the DNA-binding domain (DBD) of p73 escapes viral oncoproteins and displays an enhanced thermal stability. To further understand the variant features of p73, we solved the high‐resolution crystal structure of the p73 DBD as well as its complex with the ankyrin repeat and SH3 domains of the pro-apoptotic factor ASPP2. The p73 structure exhibits the same conserved architecture as p53 but displays a divergent L2 loop, a known site of protein–protein interaction. The loop in p73 is changed by a two-residue insertion that also induces repacking around the site of the p53 mutational hotspot R175. Importantly, the binding of ASPP2 is preserved by conformational changes in both the ankyrin repeat and SH3 domains. These results further highlight the structural variation that impacts p53 family interactions within the p53 interactome.

## Graphical abstract

**Graphical Abstract:**

**Figure:**
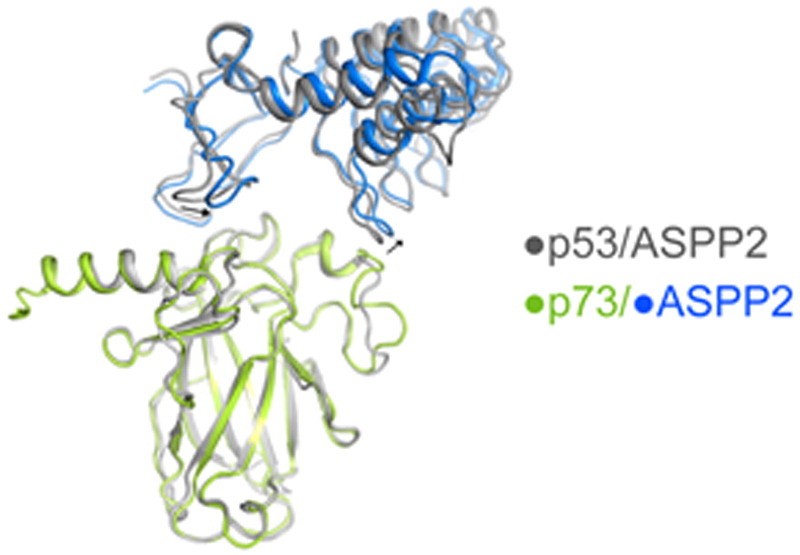

**Highlights:**

► p53 and p73 have distinct functions in development and tumor suppression. ► The 1.8‐Å structure of the p73 DBD reveals a variant L2 loop. ► The p73–ASPP2 structure shows a compensatory shift in the ASPP2 binding interface. ► Drugs have the potential to activate p73-dependent apoptosis in p53-deficient cancers.

## Introduction

The p53 family of transcription factors comprises p53 and the paralogs p63 and p73.^1^ Under conditions of cellular stress, all three proteins are activated to induce genes necessary for cell cycle arrest or apoptosis.^2^ Furthermore, p63 and p73 are required for p53-dependent apoptosis in response to DNA damage.^3^ Despite their overlapping activity, mouse knockout studies have revealed distinct functions for each member in development as well as tumor suppression. While inactivation of p53 is a common event in human cancers, it is rare for p63 or p73 to be similarly inactivated.^4^ Similarly, p53 knockout mice develop frequent spontaneous tumors,^5^ replicating Li–Fraumeni syndrome,^6^ whereas p63 and p73 null mice instead die from developmental abnormalities.^7,8^

All three proteins share a similar domain organization containing an N-terminal transactivation domain, a DNA-binding domain (DBD) and a C-terminal oligomerization domain (OD).^9^ p63 and p73 contain an additional sterile α-motif domain and an inhibitory domain at the C-terminus. Both p63 and p73 share approximately 60% sequence identity with p53 in the DBD, and DNA contact residues are strictly conserved across all three proteins.^9^

p63 and p73 occur in multiple isoforms, which have contrasting effects on tumorigenesis.^10^ The full‐length proteins (TAp63 and TAp73) include the N-terminal transactivation domain and display anti-proliferative pro-apoptotic function. In contrast, truncated transactivation‐deficient isoforms (ΔNp63 and ΔNp73) exhibit dominant-negative behavior and function as transcriptional repressors in development. Further isoforms are expressed as a result of alternative splicing.^1,10,11^ ΔNp73 has been linked to neural development and the DNA damage response pathway.^12^ The TAp73 isoforms are of particular interest as they offer a parallel tumor-suppressing function to p53. Knockout mice for TAp73 isoforms show genomic instability and a high incidence of spontaneous tumors.^13^ The TAp73 proteins activate a number of p53 target genes and share a number of common p53 activators and inhibitors.^14,15^

The activation of p73 in p53-compromised cells provides a promising and attractive anticancer strategy.^15^ A number of small molecules that promote p73-dependent apoptosis by increasing p73 expression or by extricating p73 from inhibitory complexes with mutant p53 have been identified.^16,17^ Alternatively, p73 activation has been promoted by blocking its interaction with inhibitors, such as iASPP and MDM2.^18,19^ The tumor suppressor function of p73 is lost upon its phosphorylation by the Aurora A kinase.^20^ Consequently, Aurora kinase inhibition also restores p73-dependent apoptosis in p53-deficient cells.^21^ Furthermore, the combination of nutlin-3 and VX-680 to inhibit both MDM2 and Aurora A selectively targets p53 mutant cells with reversible effects on cells expressing wild-type p53.^22^

The apoptosis‐stimulating proteins of p53, ASPP1 and ASPP2 have been shown to bind all three p53 family members and to specifically stimulate their transactivation of pro-apoptotic genes including *BAX*, *PIG3* and *PUMA*.^23,24^ The crystal structure of p53 in complex with ASPP2 has revealed a direct interaction with the p53 DBD.^25^ To address the potential for similar p73 assembly, we solved the high‐resolution crystal structure of the DBD of p73 as well as its complex with the ankyrin repeat and SH3 domains of ASPP2.

## Results

### Overview of the p73 DBD structure

The DBD (residues 112–311) of human p73 was expressed in *Escherichia coli* and purified to homogeneity using Ni-affinity and size‐exclusion chromatography. Crystals were obtained in space group *P*4_3_32 with one protein molecule in the asymmetric unit. The final p73 structure was solved using molecular replacement and refined at a resolution of 1.8 Å (see Table 1 for data collection and refinement statistics). The entire chain was traced with the exception of the three L3 loop residues from G265 to N267, which were not visible in the electron density. In addition, the side chains of residues Q133, T136, K138, Q207 and R268 were not clearly defined and were not built. Perhaps due to insufficiently reducing conditions, the protein crystallized in a somewhat oxidized state with no free cysteines (Supplementary Fig. 1).

**Fig. 1:**
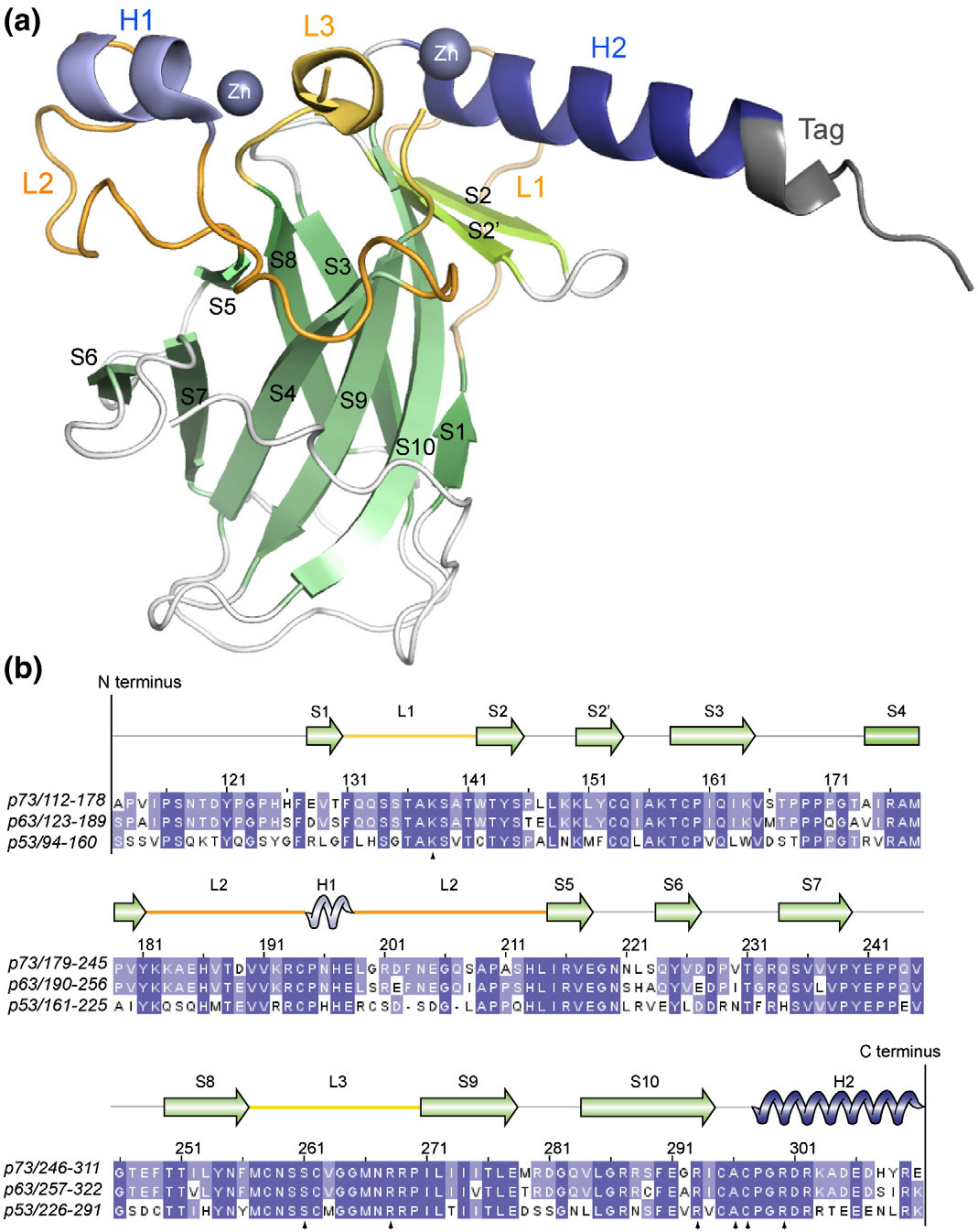
Overview of the p73 DBD structure. (a) Ribbon representation of the 1.8‐Å crystal structure of the human p73 DBD, highlighting the different secondary structural elements. The remaining portion of the C-terminal TEV cleavage site is colored gray. Bound zinc ions are shown as blue spheres. (b) Multiple sequence alignment of the p53 family DBD generated with Clustal W and visualized with JalView.^26^ Filled triangles indicate the positions of conserved DNA contact residues. Structure image was generated with PyMOL.

The p73 structure shows a conserved domain architecture, comprising a β-sandwich scaffold consisting of two antiparallel β-sheets (Fig. 1).^27^ The DNA-binding surface is constructed similarly from the large L2 and L3 loops, the S10 β-strand and a loop–sheet–helix motif that includes the L1 loop, the S2 and S2′ β-strands and the C-terminal H2 α-helix. A conserved zinc ion coordinates the L2 and L3 loops (p73 C194, H197, C258 and C262; Supplementary Fig. S1a) and contributes to the stability of the overall fold. The p73 structure also includes a second metal ion (Supplementary Fig. S1b) trapped at the interface of a crystallographic dimer formed by the H2 helix and the C-terminal His-tag (Supplementary Fig. S2). Here, the coordinating residues include the side chains of C295, C297 and D301 from one monomer and H308 from the second monomer (Supplementary Fig. S2). Both metal ions were confirmed as zinc based on multiwavelength anomalous diffraction data (Supplementary Fig. S3).

### Comparison of the p53 and p73 DBD structures

**Fig. 2:**
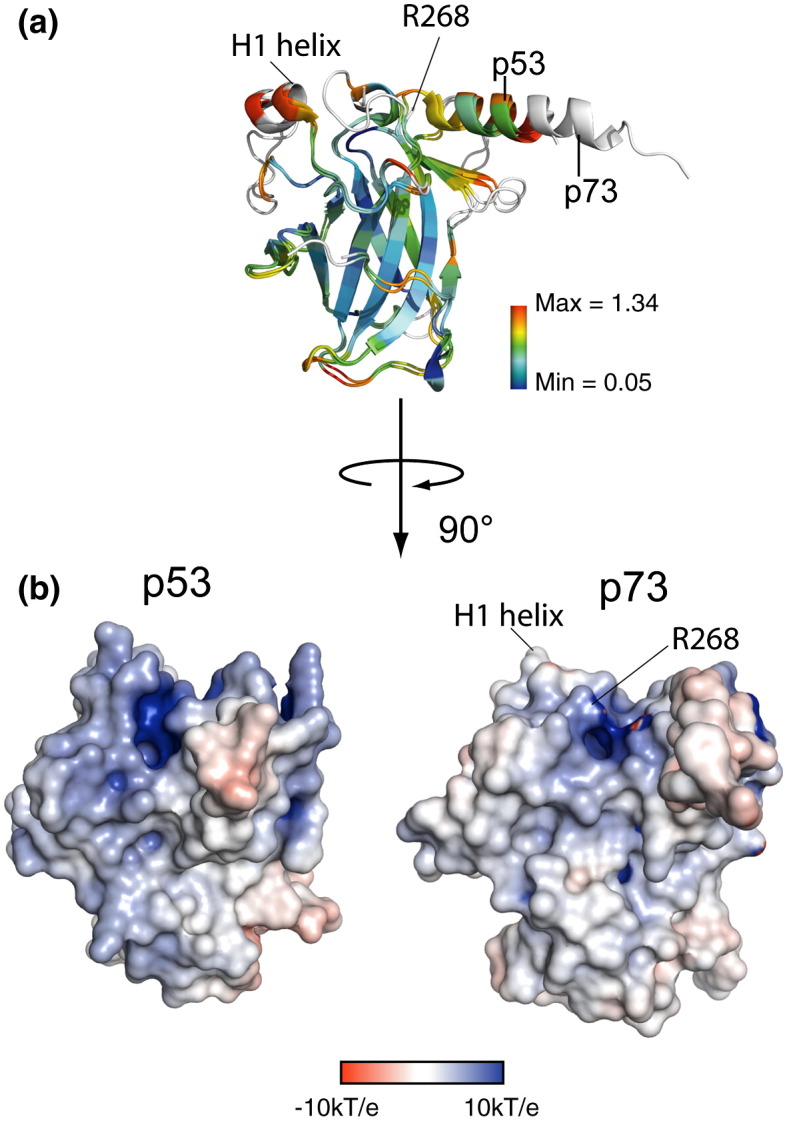
Structural comparison of p53 and p73. (a) Superposition of the p53 (PDB 2OCJ) and p73 structures in the absence of DNA colored by C^α^ RMSD. Residues where the RMSD is higher than the scale maximum are colored white. (b) Comparison of the electrostatic surface potentials of the two proteins as calculated using PyMOL and APBS.^28^ The H1 helix and R268 are labeled for orientation.

Superposition of the p53 and p73 domains reveals a high degree of structural similarity (Fig. 2a). The overall C^α^ root-mean-square deviation (RMSD) between the two structures is 1.7 Å over 191 atoms, consistent with their high sequence conservation (61% identity, Fig. 1b). However, local regions of structural deviation are apparent when the RMSD values are examined across the different secondary‐structure elements (Fig. 2a). As expected, the lowest RMSD values lie in the core β-sandwich, whereas more significant deviation occurs across the DNA binding surface. Both proteins show a strongly electropositive surface potential across the DNA contact surface but more variable charge in the L2 loop, which mediates protein–protein interactions (Fig. 2b).

**Fig. 3:**
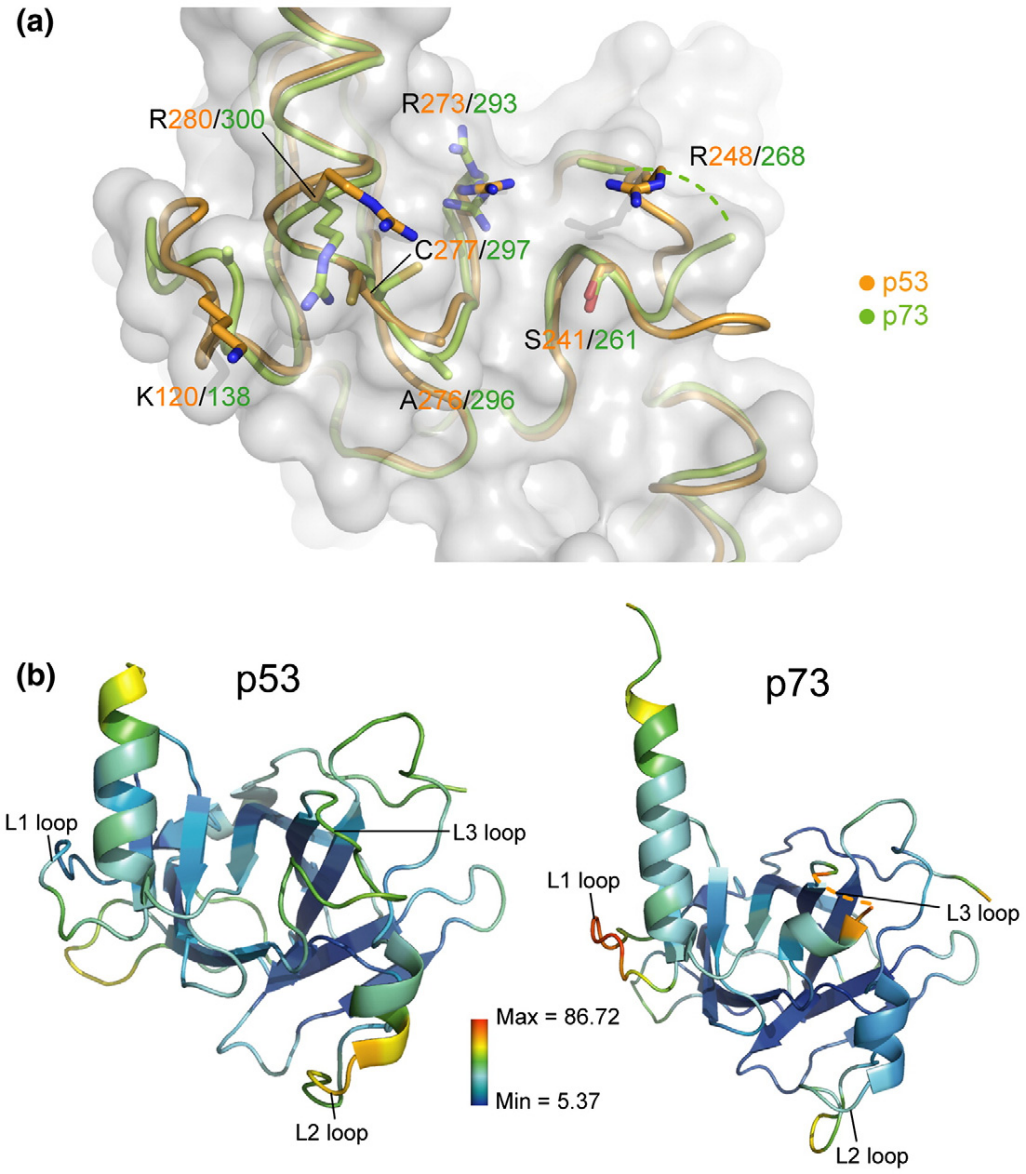
Conservation and flexibility of the DNA contact surface. (a) Superposition of the free p53 (orange) and p73 (green) structures showing conserved DNA contact residues as sticks. The side chains of p73 K138 and R268 were not visible in the electron density and were not built. A broken line indicates the disordered segment of the p73 L3 loop (G265–N267). An outline of the p73 surface is displayed in gray. (b) The structures of p53 and p73 are shown in the same orientation and colored by crystallographic *B*‐factors according to the scale shown.

DNA contact residues in p53 are strictly conserved in p73 (Fig. 3a). The side-chain conformations of p73 K138 and R268 were not defined in the electron density map, suggesting some disorder of these regions in the absence of DNA. The crystallographic *B*‐factors in p73 are indeed higher in the L1 and L3 loops (Fig. 3b). The conformations of the other flexible arginine or lysine side chains also vary between the unbound p53 and p73 structures, while p73 R293 adopts two distinct conformations. *B*-factors are lowest around the core β-sandwich, whereas the N‐ and C-termini of both p53 and p73 exhibit higher *B*‐factors and appear more flexible (Fig. 3b).

**Fig. 4:**
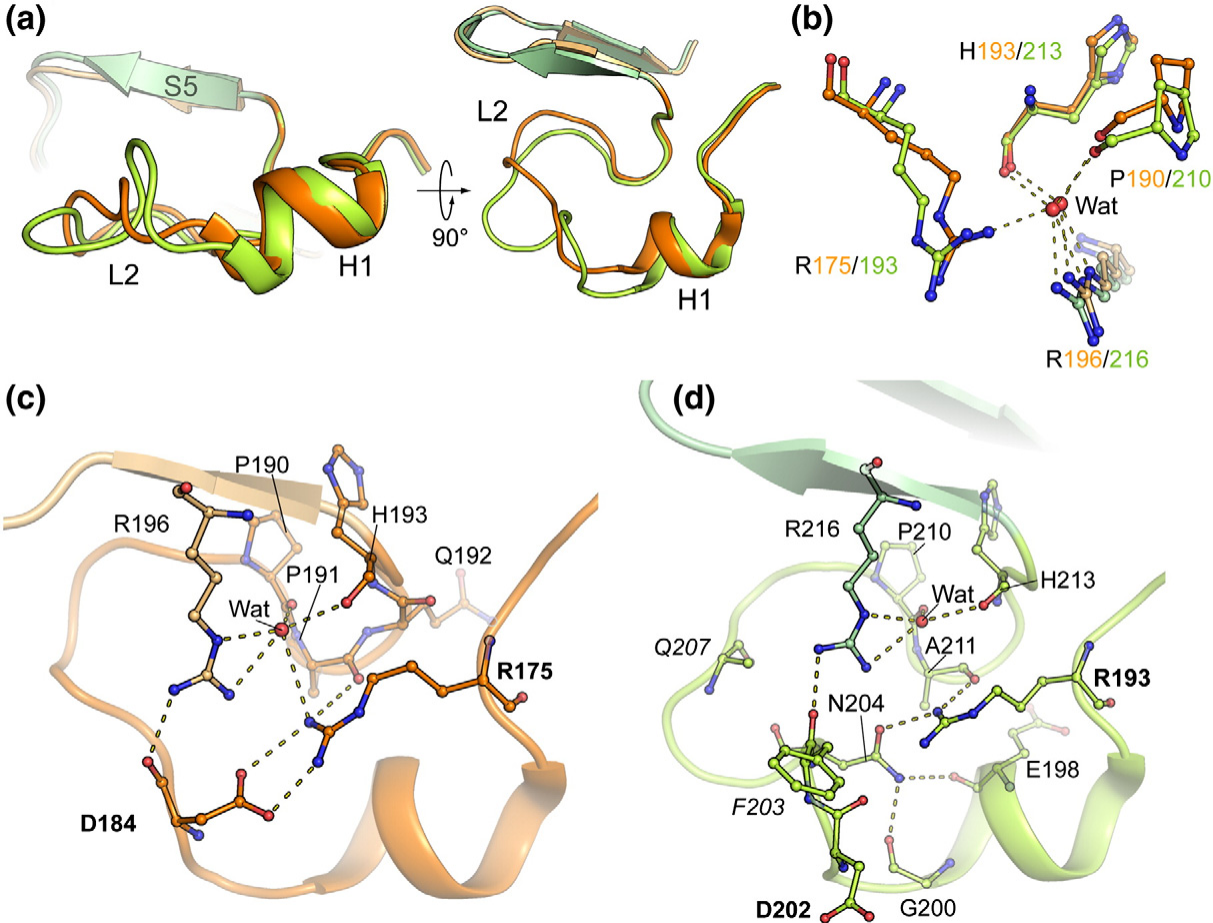
The p73 structure contains a divergent L2 loop. (a) Superposition of the free p53 (orange) and p73 (green) structures highlighting the different L2 loop conformations. (b) In both structures, conserved residues in the L2 loop form hydrogen bonds to a buried, ordered water molecule. (c) Salt bridge and hydrogen bond interactions in the p53 L2 loop. The R175 and D184 positions are highlighted. (d) The p73 L2 loop harbors a two-residue insertion (sequence alignments indicate insertion of p73 F203 and Q207 shown labeled in italics). As a result, the L2 loop structure and hydrogen bonding are changed. Most notably, the p53 R175–D184 salt bridge (equivalent to p73 residues R193 and D202 labeled in boldface) is replaced by p73 R193 interaction with N204. The N204 side chain also forms two hydrogen bonds to the H1 helix that are absent in p53.

As a consequence of their relatively high cysteine content, p53 family members have been identified as redox‐sensitive proteins.^29^ Of the 10 cysteine residues found in the p53 DBD, 7 are conserved in p73 (Supplementary Fig. S1). Unusually, a disulfide bond is observed in the p73 structure between C153 (S2′) and C159 (S3), although the main‐chain atoms remain similarly placed to p53 C135 and C141 (Supplementary Fig. S1c), suggesting that the oxidation has limited structural significance and is most likely the result of insufficiently reducing conditions during purification. The same site in p53 has a third cysteine residue C124 (β-strand S2) in close proximity, which may increase the propensity for p53 misfolding upon oxidation. The two remaining cysteine substitutions include p73 F249 and G200, which replace p53 C229 and C182, respectively. The smaller substitution of G200 stabilizes the p73 H1 helix in a distinct conformation of the L2 loop that facilitates hydrogen bond formation between G200 and N204 (Fig. 4a and d).

### The L2 loop is structurally divergent across the p53 family

The most divergent region in the DBD is identified as the L2 loop (p73 residues 182–212). Here, the sequence conservation with p53 is weak, especially in the region C-terminal to the H1 helix, where p73 and p63 contain a two-residue insertion (Fig. 1b). As a result, p73 residues R201–A209 adopt a markedly different conformation to p53 C182–A189 (Fig. 4a). This region corresponds to the most solvent-exposed part of the L2 loop and is anchored to the core β-structure by two conserved arginine residues, R175 (L2) and R196 (S5) in p53 (R193 and R216 in p73). These side chains pack closely to within 3.5 Å and adopt similar conformations in the two structures (Fig. 4b). A conserved water molecule is bound at their interface, facilitating additional hydrogen bond opportunity with the carbonyls of p53 P190 and H193 (or p73 P210 and H213) (Fig. 4b). In p53, the most critical interactions formed by the two arginines are with D184, including a salt bridge from R175 and a hydrogen bond from R196 (Fig. 4c). Consequently, the tumorigenic mutation R175H has been identified as the most disruptive for p53 folding.^30^

Despite the similar positions of p73 R193 and R216 and the conservation of the D184-aligned residue (p73 D202), the equivalent interactions are not observed in the p73 structure (Fig. 4d). Unexpectedly, the D202 side chain of p73 extends away from the L2 loop into solvent. Instead, the p73 insertion induces a loose helical turn that places N204 in the equivalent position to hydrogen bond with both p73 R193 and R216. By occupying the center of the loop, N204 also lies within hydrogen‐bonding distance of the backbone oxygens of E198 and G200 in the H1 helix (Fig. 4d). The loop conformation in p73 is further stabilized by main‐chain hydrogen bonding (not shown): the carbonyl of R201 contacts the backbone amide of N204 and E205, while the backbone carbonyl of N204 contacts the backbone amide of Q207. Perhaps as a result of these additional hydrogen bond interactions, the *B*‐factors in the H1 helix of p73 are lower than those in p53 (Fig. 3b).

### Molecular consequences for p73–ASPP2 interaction

**Fig. 5:**
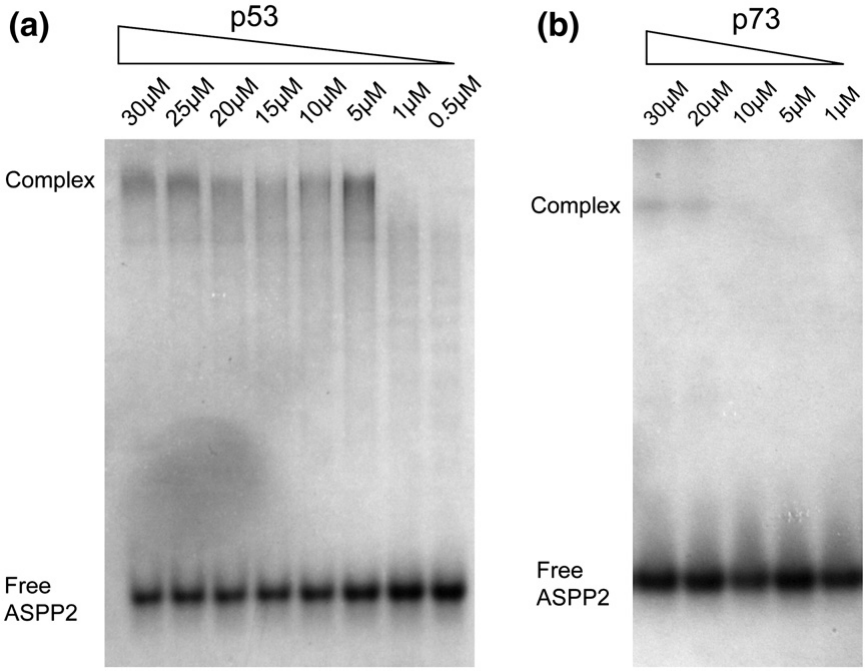
Native-gel mobility shift assay for ASPP2 binding. (a) Binding reactions contained 20 μM ASPP2 and decreasing concentrations of p53 DBD as indicated (p53 amino acids 94–312 were purified as described previously^31^). Binding was performed at 37 °C for 5 min followed by 30 min of incubation on ice. Proteins were buffered in 50 mM Tris, pH 7.2, 50 mM NaCl, and 5 mM DTT. Complexes were separated from unbound ASPP2 on a 10% polyacrylamide gel in Tris–glycine buffer at pH 8.3 and visualized with InstantBlue Coomassie stain. The p53 DBD has a net positive charge at this pH and does not enter the gel. (b) Under similar conditions, binding of the p73 DBD was not observed. A screen of different buffer conditions identified binding in 50 mM sodium phosphate, pH 7.2, 50 mM NaCl, 5 mM DTT, 50 mM l-arginine, and 50 mM l-glutamate. Binding reactions contained 20 μM ASPP2 and p73 amino acids 112–315 at decreasing concentrations as indicated. Binding was performed at 4 °C overnight and native PAGE was conducted as above.

**Fig. 6:**
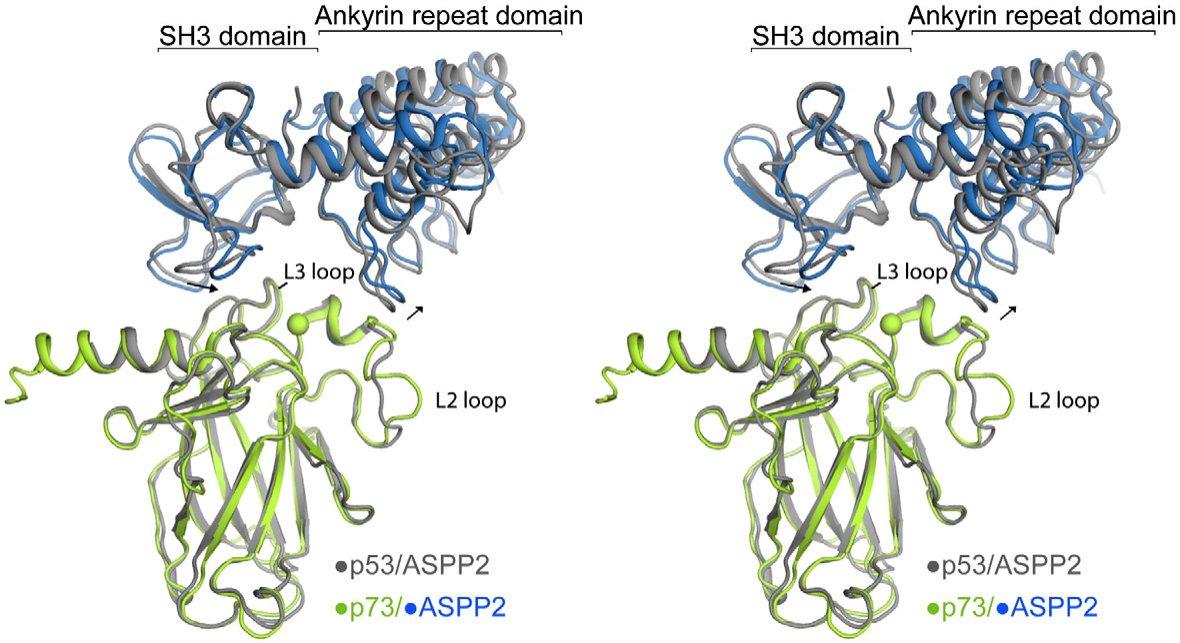
Structural overview of the p73–ASPP2 complex. Stereo view of the p73–ASPP2 complex (green/blue) superimposed on the equivalent p53 structure (gray). Arrows highlight a subtle shift in the ASPP2 position. The single zinc ion associated with p73 is shown as a green sphere.

The H1 helix and surrounding L2 loop do not participate directly in DNA interaction but contribute instead to dimer contacts in the DNA-bound tetramer (Supplementary Fig. S4a), as well as other protein–protein interactions (Supplementary Fig. S4b). Superposition of the free p73 structure with the p53–ASPP2 complex suggests a potential steric clash from the p73 H1 helix (Supplementary Fig. S4b). Binding of ASPP2 to p53 and p73 DBD was confirmed in the low micromolar range by native-gel mobility shift assay (Fig. 5). Interestingly, the binding of p73 appeared slightly less stable than that of the p53 complex. To investigate the p73 interaction further, we tested a variety of expression constructs in co-crystallization. Due to dissociation of p73–ASPP2 complexes during preparative gel filtration (Supplementary Fig. S5), the two proteins were purified separately and then mixed at a 1:1 molar ratio prior to crystallization. Crystals of the complex were obtained in space group *I*121 when combining the p73 DBD with the ankyrin repeat and SH3 domains (residues 891–1128) of human ASPP2. The structure of the complex (Fig. 6) was solved using molecular replacement and refined at a resolution of 2.6 Å (see Table 1 for data collection and refinement statistics). The six p73 chains in the asymmetric unit were traceable between residues 114 and 311. Bound ASPP2 chains were traceable between residues 920 and 1121 and showed minor structural deviations in the SH3 RT and n-Src loops, as well as the protein termini. A full description of the crystallized ASPP2 fold (also known as 53BP2) has been reported previously and comprises four ankyrin repeats and a C-terminal SH3 domain.^25^

**Fig. 7:**
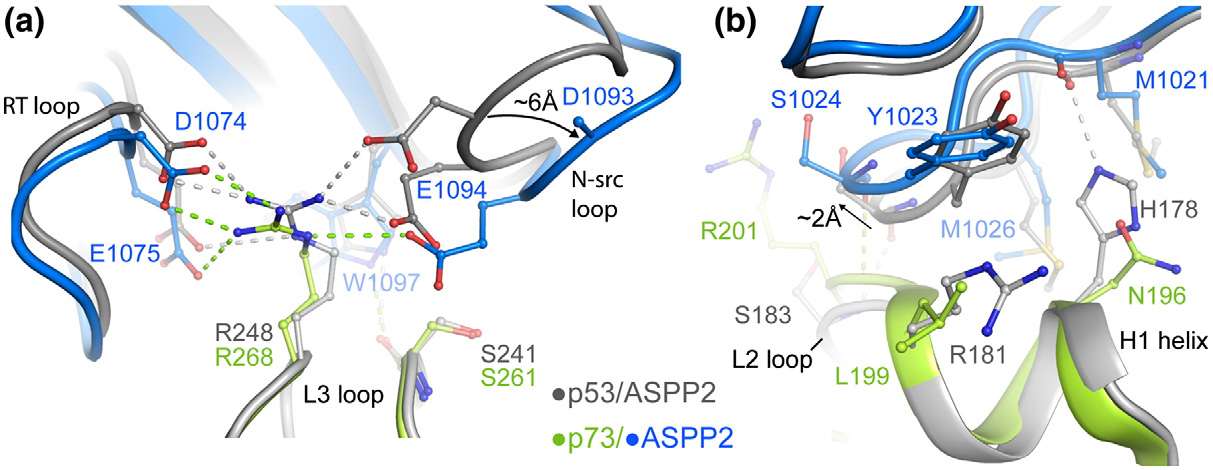
Specific packing interactions in the p73–ASPP2 complex. (a) Superposition of the p53 and p73 complexes reveals a significant shift in the position of the n-Src loop, leading to the loss of p73 R268 interaction with ASPP2 D1093 (this side chain was not clearly defined in the electron density). (b) Structural superposition again reveals a shift in the packing of the p73–ASPP2 complex. The L2 loop insertion in p73 induces a subtle change in the position of the H1 helix. Consequently, the loop of the fourth ankyrin repeat in ASPP2 is shifted by ~ 2 Å to avoid steric clashes. Significant sequence changes result in distinct packing interactions in the two complexes.

Overall, the p73–ASPP2 structure is highly conserved with the p53 complex but exhibits a shift in the ASPP2 position to better accommodate the variant p73 L2 loop (Fig. 6). As a result, the contact surface area of the p53–ASPP2 structure (753 Å^2^) is slightly greater than that of the p73–ASPP2 complex, which varies across the noncrystallographic‐symmetry‐related molecules from 672 to 742 Å^2^. The binding of both proteins is directed primarily by the L3 loop interaction with the ASPP2 SH3 domain (Fig. 7a) but supported by further L2 loop contact with the fourth ankyrin repeat (Fig. 7b).

The p53 family proteins represent atypical SH3-binding proteins, binding through two separate L3 loop segments rather than a single extended peptide (Fig. 7a). The most significant contact is made by the cancer hotspot residue p53 R248 and the equivalent p73 residue R268, which was disordered in the unbound p73 structure. Both side chains undergo rearrangements to fill the large SH3 pocket formed between the RT and n-Src loops (Supplementary Fig. S6). Comparison of the two structures reveals that the n-Src loop in the p73 complex is moved ~ 6 Å away from its position in the equivalent p53 structure (Fig. 7a). Perhaps as a consequence, p73 R268 is oriented more towards the RT loop than p53 R248. Thus, while both residues bind ASPP2 D1074, E1075 and E1094, the p73 R268 side chain has lost a further contact with D1093. In the p73 complex, the n-Src loop movement also removes a hydrogen bond contact made by p53 R280 (H2) (not shown). Other SH3 interactions in the p73 complex are generally conserved, such as the hydrogen bond between p73 S261 and ASPP2 W1097 (Fig. 7a). However, hydrophobic contact by p53 M243 is reduced with the smaller p73 V263 (not shown).

The L2 loop interaction site is more divergent. To avoid a steric clash with p73 L199–R201, the fourth ankyrin repeat of ASPP2 is moved by ~2Å relative to the p53 complex and there is an ~180° change in the Ψ-angle of ASPP2 Y1023. A main‐chain hydrogen bond between p73 R201 and ASPP2 S1024 is maintained, as well as the van der Waals interactions with ASPP2 M1026 (Fig. 7b). However, the only side‐chain hydrogen bond from p53 H178 to the backbone carbonyl of M1021 is lost with the substitution of p73 N196 (Fig. 7b).

## Discussion

The p53 family DBD has undergone considerable structural evolution, as evidenced by the distinct structure of the earliest known orthologue, *Caenorhabditis elegans* Cep-1.^32^ An important finding from the structure determination of the human p73 DBD is the apparent lack of structural evolution following the expansion of the ancestral *p53* gene into the three family members p53, p63 and p73. In comparison, the C-terminal OD has evolved structural mechanisms that restrict p53 assembly with p63 and p73.^33–35^ The conservation of the DBD fold in humans is consistent with the similar consensus DNA response elements determined for p53, p63 and p73.^36,37^ Together, these data highlight the overriding importance of DNA binding to p53 family activities.

Despite the overall conservation, we identified significant structural changes in the p73 L2 loop. Here, sequence alignments and comparative modeling would predict the strict conservation of the p53 R175–D184 salt bridge, which is critical for p53 folding as shown by the tumorigenic hotspot mutation R175H. It is therefore highly surprising that this interaction is changed in p73. Instead, p73 harbors a two-residue insertion that extends the L2 loop and facilitates a more widely distributed network of hydrogen bonding. A similar L2 loop arrangement is observed in the recently solved crystal structures of the p63–DNA complex (Supplementary Fig. S7).^38^ These structural changes are likely to contribute to the enhanced thermodynamic stability of p63 and p73 relative to p53.^39^

The tumor suppressor ASPP2 binds to all three p53 family members and specifically up-regulates their pro-apoptotic function.^24^ The involvement of p63 and p73 is confirmed in p53 null cells by RNA interference.^24^ Although the binding mode of p73 is similar to that of the p53–ASPP2 complex, we show that it requires additional structural rearrangements in ASPP2 to avoid steric clashes with the H1 helix, which bulges out from p73 due to its L2 loop insertion. We observe shifts in the positions of both the n-Src loop and the fourth ankyrin repeat that alter hydrogen bond interactions and reduce slightly the overall buried surface area of the complex. Initial estimates of p53–ASPP2 binding using BIAcore (*K*_d_ = 30 nM)^25^ and solid‐phase ELISA (*K*_d_ = 23 nM)^40^ are likely to overestimate the true affinity of the interaction as suggested by isothermal titration calorimetry measurements of all three p53 family members (*K*_d_ = 2 to 5 μM).^41–43^ Such affinities are consistent with our own native-gel mobility shift assays (Supplementary Fig. 7) as well as the ASPP2 crystal structures, which are scored poorly by the Protein Interfaces, Surfaces and Assemblies server, indicating that these structures are not recognized as typical high‐affinity protein–protein complexes.^44^

**Fig. 8:**
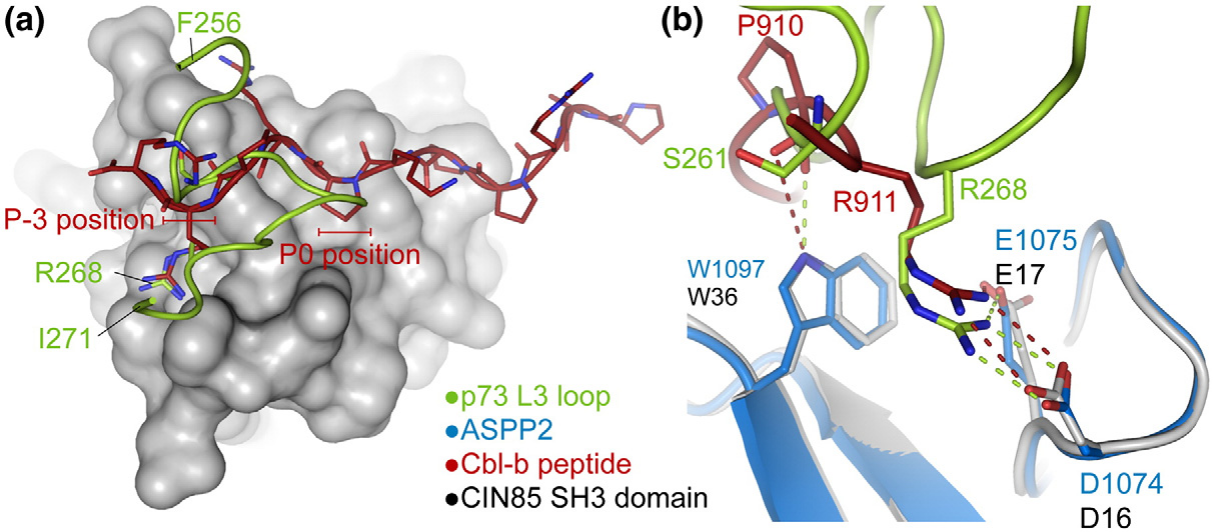
Conserved ligand interactions with the RT loop. (a) Superposition of the p73–ASPP2 complex with a Cbl-b peptide bound to the SH3 domains of CIN85 (PDB ID 2BZ8).^45^ The surface of one CIN85 SH3 domain is shown in gray, and the Cbl-b and p73 chains are shown in red and green, respectively. The p73 L3 loop is structurally distinct from the extended proline-rich sequences of most SH3 ligands. (b) Although the binding mode of p73 is distinct from extended SH3 ligand interactions, several features are conserved. These include the critical arginine interaction with the RT loop and a backbone hydrogen bond to ASPP2 W1097.

The low micromolar affinity of the ASPP2 complexes remains in the normal range for SH3 domain interactions. Typical proline-rich ligands form backbone interactions across the SH3 surface, while an arginine residue orients their binding motif by interacting with the RT loop. Although the binding mode of p53 family members is distinct, interacting through two separate loop regions, several features are conserved (Fig. 8a). The RT loop interaction of p73 R268 shows a highly similar bonding configuration to Cbl-b R911 in complex with the endocytic regulator protein CIN85 (Fig. 8),^45^ although the backbone positions are quite different (Fig. 8b). The backbone hydrogen bond formed by p73 S261 is also analogous to the interaction of the P_− 2_ residue of classical SH3 binding motifs (Fig. 8b). The interaction between Cbl-b and CIN85 is itself unusual as the proline–arginine motif of Cbl-b spans across the multiple SH3 domains of CIN85.^45^

The p73 DBD is the last globular domain in the p53 family to be structurally determined. Its structure reveals a conserved fold for DNA binding but a divergent L2 loop. While the binding of ASPP2 is maintained, some other known interacting partners of p53 DBD are lost, most notably viral oncoproteins such as the SV40 large T-antigen. The structure of this p53 complex suggests no obvious sequence requirements to account for this specificity but reveals a dramatic conformational change in the p53 DNA-binding surface.^46^ Potentially, the enhanced thermodynamic stability and variant structure of p73 may disfavor this structural rearrangement and therefore inhibit binding. A common model emerges from the structural and functional data supporting the overlapping but nonredundant roles of p53 family members in development and tumor suppression. Pharmacological manipulation of the pro-apoptotic functions of p73 and ASPP2 presents an attractive target for tumor suppression in p53-deficient cancers.

## Materials and Methods

### Plasmids

The DBD of human p73 (residues A112–E311; UniProt accession number O15350) and the DBD and OD of p73 (residues A112–S400) were cloned into the plasmid pNIC-CTHF. The ankyrin and SH3 domains of human ASPP2 (residues P891–A1128; UniProt accession number Q13625) were cloned into the plasmid pCOEX-LIC1. The pNIC-CTHF vector provides a C-terminal hexahistidine and FLAG tag, preceded by a tobacco etch virus (TEV) protease A cleavage site, whereas the pCOEX-LIC vector provides an N-terminal hexahistidine tag followed by a TEV cleavage site.

### Protein expression and purification

All proteins were expressed separately in *E. coli* BL21(DE3) R3 pRARE cells, where R3 denotes a derivative of BL21(DE3) resistant to a strain of T1 bacteriophage (Structural Genomics Consortium Oxford) and the pRARE plasmid originates from the Rosetta strain (Novagen). Bacterial cells were cultured at 37 °C in LB media supplemented with 50 μg ml^− 1^ kanamycin and 34 μg ml^− 1^ chloramphenicol for p73 or only 34 μg ml^− 1^ chloramphenicol for ASPP2. At mid-log phase, expression was induced by addition of 0.2 mM IPTG and incubated overnight at 18 °C. Cells were harvested by centrifugation and resuspended in binding buffer (50 mM Hepes, pH 7.5, 500 mM NaCl, 5% glycerol, and 5 mM imidazole). Resuspended pellets were stored at − 20 °C. Thawed cell pellets were supplemented with 1 mM PMSF and 0.5 mM tris(2-carboxyethyl)phosphine (TCEP) and disrupted by sonication (p73) or using an Emulsiflex C3 homogenizer (ASPP2). Cell extracts were clarified by centrifugation and DNA was removed using a diethylaminoethyl cellulose (Whatmann) column. His-tagged proteins were purified under gravity flow using nickel-Sepharose (GE Healthcare) columns. Bound proteins were washed in binding buffer containing 30 mM imidazole and eluted in a stepwise gradient with binding buffer containing 50, 100, 150 or 250 mM imidazole. Eluted proteins were supplemented with 5 mM dithiothreitol (DTT) and treated with TEV protease overnight at 4 °C. The ASPP2 protein was diluted 10-fold and further purified using a 5‐ml HiTrap Q HP ion‐exchange column (GE Healthcare). Finally, all proteins were purified on a HiLoad 16/60 Superdex 200 Prep Grade column using an ÄKTAxpress system (GE Healthcare). The correct mass of each protein was confirmed by electrospray ionization mass spectroscopy.

### Crystallization

All crystallization was performed using the sitting drop vapor diffusion method. The p73 DBD was buffered in 50 mM Hepes, pH 7.5, 300 mM NaCl, and 0.5 mM TCEP and concentrated in a 10‐kDa‐cutoff Amicon Ultra-15 concentrator (Millipore) to 19 mg ml^− 1^, assuming a molar extinction coefficient of 18,910 M^− 1^ cm^− 1^. Crystals were grown at 20 °C in 150‐nl sitting drops mixing 50 nl of protein solution with 100 nl of a reservoir solution containing 1.2 M sodium potassium tartrate, 0.25% polyethylene glycol monomethyl ether 5000 and 0.1 M Tris, pH 9. On mounting, p73 crystals were cryo-protected with an additional 20% glycerol before vitrification in liquid nitrogen. The p73–ASPP2 complex was prepared by mixing the purified proteins at a 1:1 molar ratio. The complex buffered in 10 mM Hepes, pH 7.5, and 0.5 mM TCEP was concentrated to 11 mg ml^− 1^, assuming an extinction coefficient of 58,330 M^− 1^ cm^− 1^. Crystals were grown at 4 °C in 150‐nl sitting drops mixing 100 nl of protein solution with 50 nl of a reservoir solution containing 1.0 M sodium–potassium phosphate and 0.1 M acetate, pH 4.5. On mounting, crystals were cryo-protected with an additional 20% ethylene glycol.

### Structure determination and refinement

The p73 DBD diffraction data were collected at the Diamond Light Source beamline I02. Data processing and refinement were carried out using the CCP4 suite of software.^47^ Data were integrated with Mosflm^48^ and scaled with SCALA.^49^ Phases were calculated by molecular replacement in Phaser^50^ using the structure of the unbound p53 DBD as a search model [Protein Data Bank (PDB) ID 20CJ].^51^ The initial model was improved using iterative rounds of manual model building in Coot^52^ and restrained refinement in REFMAC5.^53^ During later stages of refinement, translation/liberation/screw (TLS) parameters were introduced as calculated by the TLS motion determination server.^54^

The p73–ASPP2 diffraction data were collected at the Diamond Light Source beamline I03. Data were integrated with XDS^55^ and scaled with SCALA.^49^ Phases were calculated by molecular replacement in Phaser^50^ using the structure of the p73 DBD (PDB ID 2XWC) and the structure of the ankyrin repeat and SH3 domains of ASPP2 (PDB ID 1YCS, chain B)^25^ as search models. The crystal asymmetric unit contained six p73 and six ASPP2 chains. The initial electron density map was improved using PARROT^56^ and the model was rebuilt using Buccaneer.^57,58^ The model was improved and refined using iterative rounds of manual model building in Coot^52^ and restrained refinement in REFMAC5.^53^ During later stages of refinement, TLS parameters were introduced as calculated by the TLS motion determination server.^54^ The last stages of refinement were carried out with BUSTER,^59^ using the TLS group definitions determined above and automatically determined noncrystallographic symmetry restraints.^60^ Structures were validated using the Joint Center for Structural Genomics quality control server†. Structure analysis was performed using PyMOL.^61^ Data collection and refinement statistics are presented in Table 1.

### Multiwavelength anomalous diffraction

Three data sets were collected from a second p73 DBD crystal to obtain anomalous maps that would distinguish between zinc and other potential metal ligands. Data were collected at 12.658, 8.856 and 7.999 keV. The anomalous scattering coefficients (*f*″) of zinc and nickel at these energies are presented in Supplementary Table S1. Values were obtained from the website of the Biomolecular Structure Centre, University of Washington, Seattle.^2^ Data were processed as described above for p73 DBD and phased using the refined p73 structure as a molecular replacement solution in Phaser.^50^ Fast Fourier transform was used to generate anomalous maps based on the low‐, medium‐ and high‐energy data sets and a peak search performed using the integrated PEAKMAX functionality. Strong peaks were identified at both metal-ion binding sites in high-energy anomalous maps with a height of 13.27 σ and 12.01 σ. The next highest peak was 4.37 σ. Both peaks disappeared on examination of the medium- and low-energy anomalous maps, indicating that zinc ions were coordinated at both sites (see Supplementary Fig. S3).

### Accession numbers

Coordinates and structure factors have been deposited in the PDB with accession numbers 2XWC and 4A632XWC4A63.

## Figures and Tables

**Table 1:** Data processing and refinement statistics

*Data collection*		
PDB ID	2XWC	4A63
Space group	*P*4_3_32	*I*121
Cell dimensions		
*a*/*b*/*c* (Å)	110.4/110.4/110.4	132.8/170.1/177.6
α/β/γ (°)	90/90/90	90/92/90
Resolution (Å)	1.82 (1.92–1.82)	2.65 (2.79–2.65)
Unique observations	21,247 (3017)	111,026 (16,378)
Completeness (%)	100 (100)	97.4 (98.7)
Redundancy	13.2 (12.9)	3.7 (3.8)
*R*_merge_	0.165 (1.239)	0.115 (0.686)
*I*/σ*I*	11.9 (2)	8.4 (2.1)

*Refinement*		
Resolution (Å)	1.82	2.65
*R*_work_/*R*_free_ (%)	0.1890/0.2282	0.2151/0.2441
Number of atoms		
Protein	1590	18,380
Heteroatoms	19	46
Water	175	806
*B*‐factors		
Protein	24.64	51.32
Heteroatoms	39.97	57.34
Water	28.7	43.76
RMSD		
Bond lengths (Å)	0.016	0.01
Bond angles (°)	1.542	1.08

Numbers in parentheses refer to the highest-resolution shell.

## Footnotes

† http://smb.slac.stanford.edu/jcsg/QC/

‡ http://skuld.bmsc.washington.edu/scatter/AS_periodic.html
