# Supplementary material for: Structural Basis for ASPP2 Recognition by the Tumor Suppressor p73
Source: J Mol Biol. 2012 Nov 2;423(4):515–27. doi: 10.1016/j.jmb.2012.08.005 (PMC3472557; doi:10.1016/j.jmb.2012.08.005)
Supplement: Supplementary file 1 — Supplementary data [file mmc1.pdf]

Supplementary Material

Structural basis for ASPP2 recognition by the tumour suppressor p73

Peter Canning, Frank von Delft and Alex N. Bullock*

Structural Genomics Consortium, University of Oxford, Old Road Campus, Roosevelt Drive, Oxford OX3 7DQ, UK

*Corresponding author:

Alex N. Bullock; email: alex.bullock@sgc.ox.ac.uk; Address: Structural Genomics Consortium, University of Oxford, Old Road Campus, Roosevelt Drive, Oxford OX3 7DQ, UK; Tel: +44 (0)1865 617754; Fax: +44 (0)1865 617575.

λ (Å)	E (KeV)	*f*'' (Zn)	*f*'' (Ni)
0.9795	12.685	2.45	1.95
1.4	8.865	0.59	3.5
1.55	7.999	0.7	0.5

Table S1 – Anomalous scattering coefficients of zinc and nickel (in electrons) at the wavelengths used for data collection during the MAD experiment. Values retrieved from the website of the Bimolecular Structure Centre, University of Washington, Seattle (http://skuld.bmsc.washington.edu/scatter/AS_periodic.html).

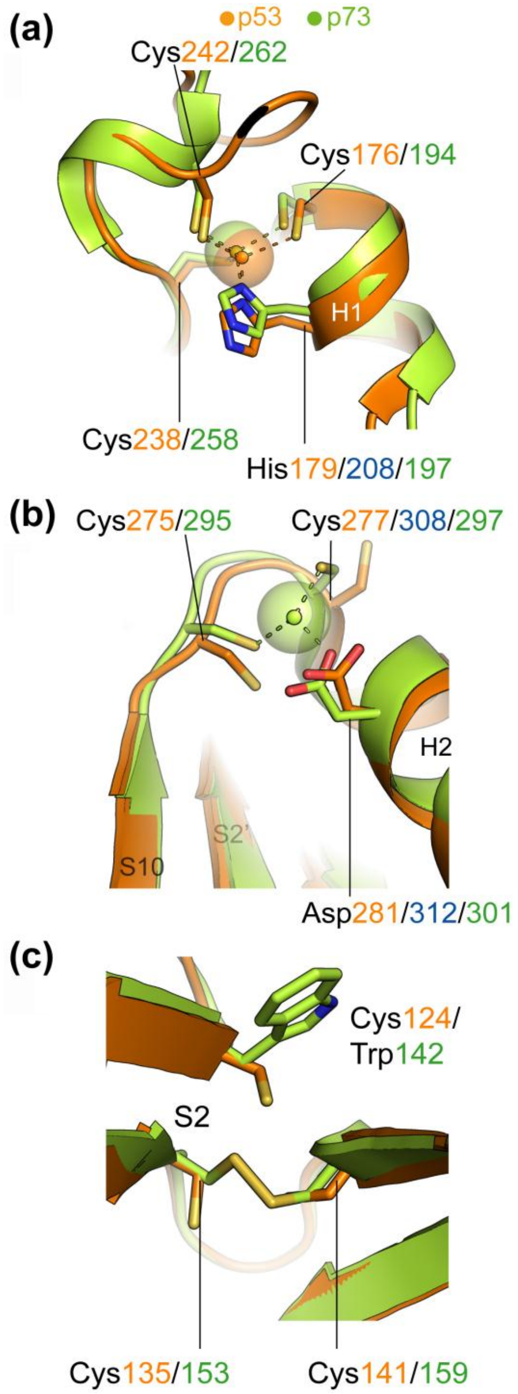

Figure S1. Cysteine residue interactions in the p73 DBD structure. The p73 DBD crystallized in a slightly oxidized state in which all seven cysteine residues were either complexed with a metal ion or part of a disulfide bond. **(a)** Comparison between the conserved

zinc binding sites of p53 (orange) and p73 (green). Proteins are shown in ribbon representation with the side chains of the four zinc-coordinating residues shown as sticks and labelled. Bound zinc ions are shown as spheres and colored according to their protein complex. **(b)** A second zinc ion was bound at the surface of the p73 DBD structure. The coordinating residues from one p73 chain are shown together with the equivalent site of p53. The metal ion was trapped at the interface of a crystallographic dimer and coordinated also by H308 from the neighbouring p73 chain (see Supplementary Fig. S2). **(c)** The remaining two cysteines in the p73 DBD structure formed a disulfide bridge that was not previously observed in a p53 family member. Superposition with p53 revealed that there were no resulting structural pertubations, but that the sensitivity of p53 to oxidation may be increased by presence of the proximate cysteine p53 C124, which in p73 is replaced by W142. The disulfide and surface zinc shown in **c** and **b** were not present in the p73-ASPP2 complex.

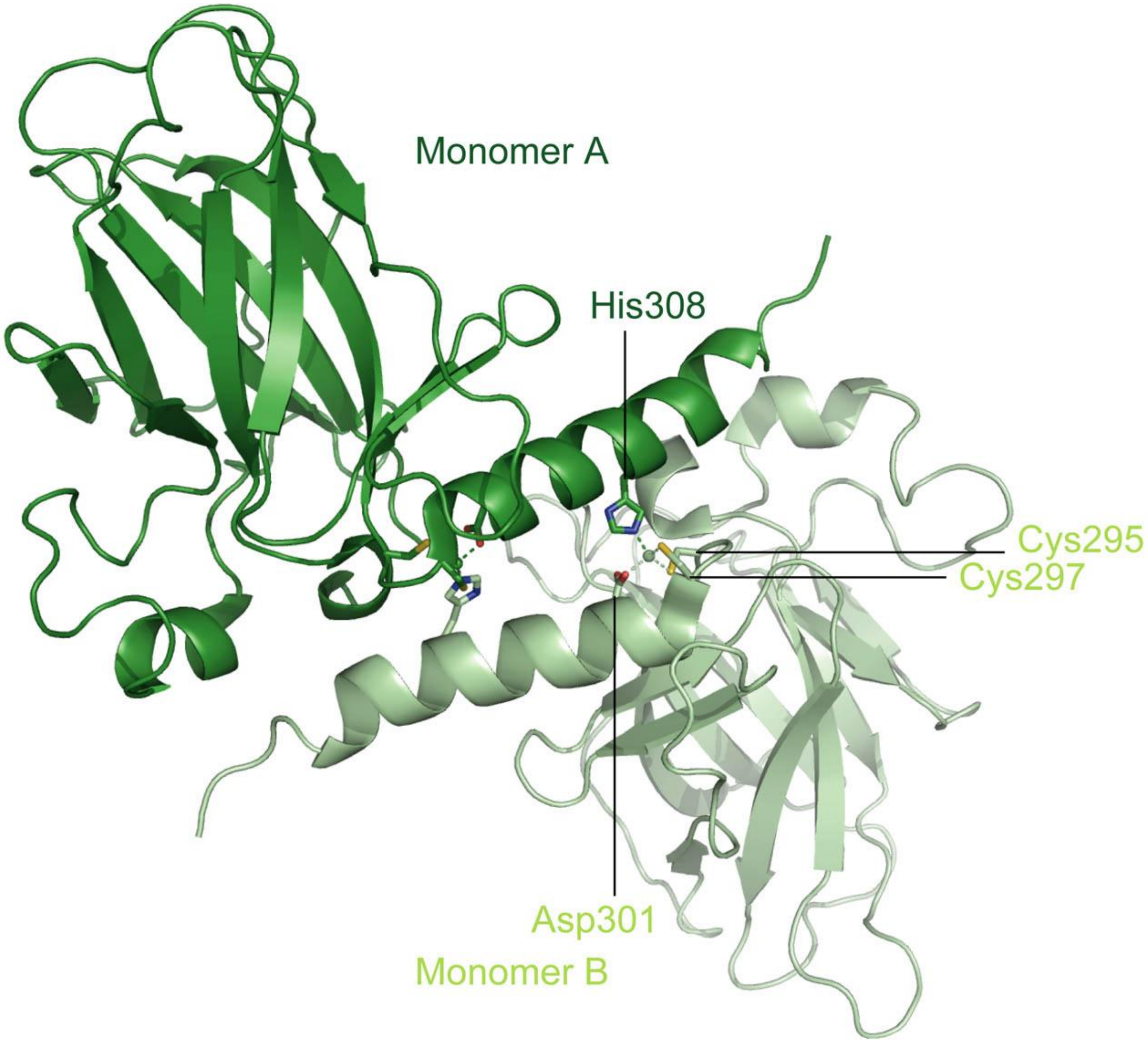

Figure S2. Metal ions contribute to the formation of a crystallographic p73 dimer. The crystal packing is shown for two neighboring p73 chains. The second zinc ion associated with each chain in Supplementary Fig. S1b is shown as a sphere and bound at the dimer interface. Coordinating residues are shown in stick representation and include C295, C297 and D301 from one chain and H308 from the second. The coordinating residues are conserved in p53 with the exception of H308 which is replaced by an asparagine.

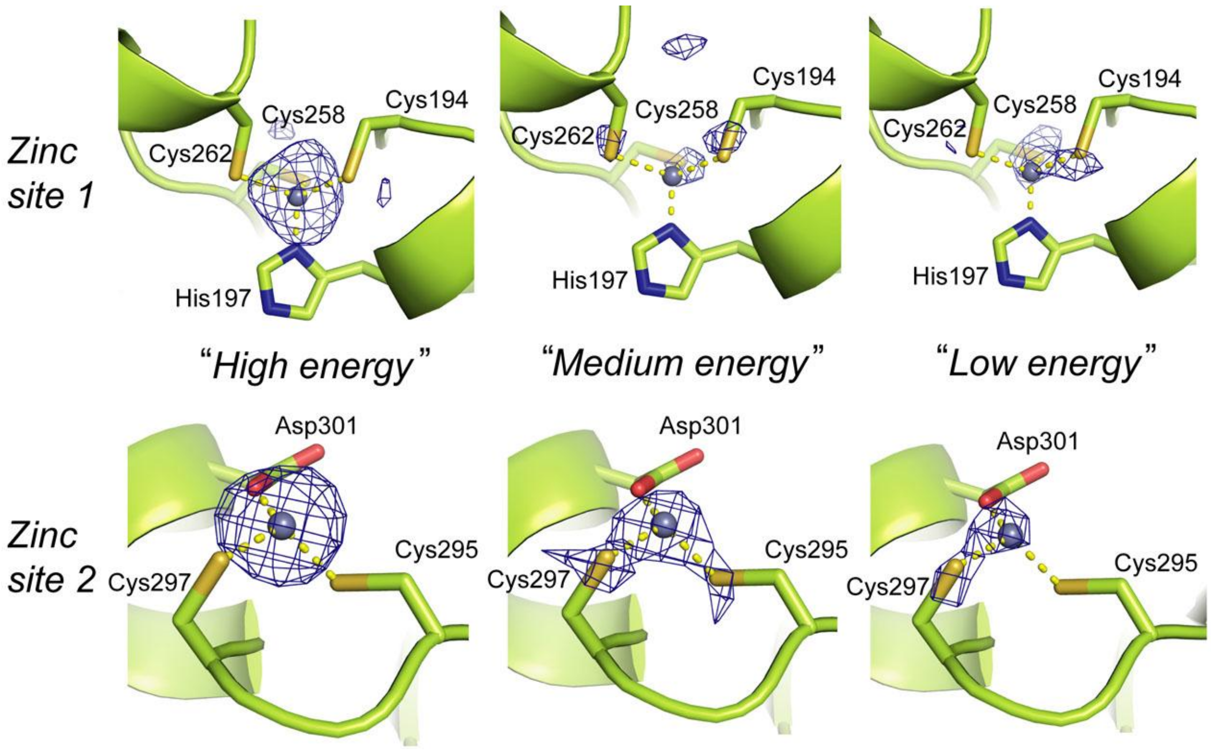

Figure S3. Electron density derived from anomalous difference maps confirms the bound metal ions as zinc. Maps (blue mesh) are calculated with anomalous differences measured at high, medium and low energy, respectively, and are displayed contoured to 3σ for zinc site 1 (conserved across the p53 family) and zinc site 2 (unique to p73 DBD crystal packing). Coordinating residues are displayed as sticks and labelled.

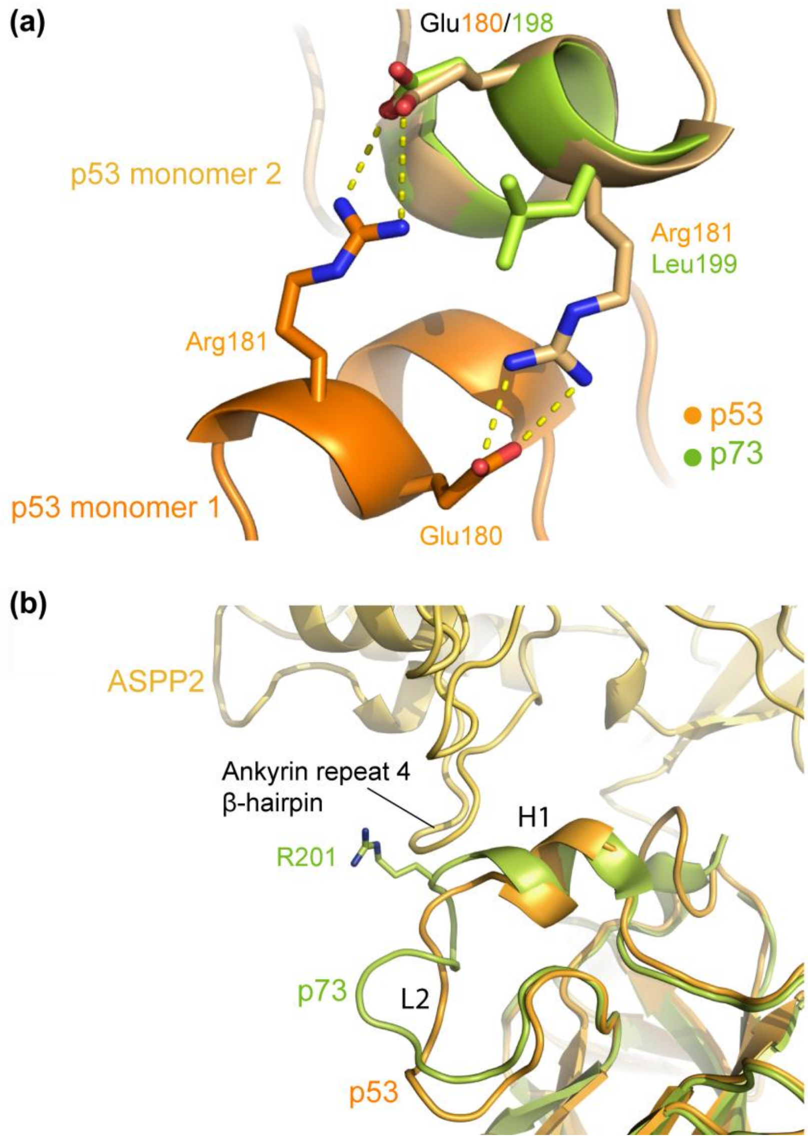

Figure S4. Changes in the p73 L2 loop and H1 helix are predicted to destabilize the intermolecular interactions of p53. (a) The H1 helix of p53 (orange) mediates dimer contacts in the DNA-bound p53 tetramer through the salt bridges of E180 and R181 (shown as dashed lines). Superposition of a p73 DBD chain (green) shows that salt bridges cannot form in an equivalent p73-DNA complex due to the substitution of p53 R181 with p73 L199. **(b)** Superposition of the p73 DBD and the p53-ASPP2 complex (PDB ID 1YCS) indicates that structural changes in the interface are necessary to avoid a steric clash with the fourth ankyrin repeat of ASPP2.

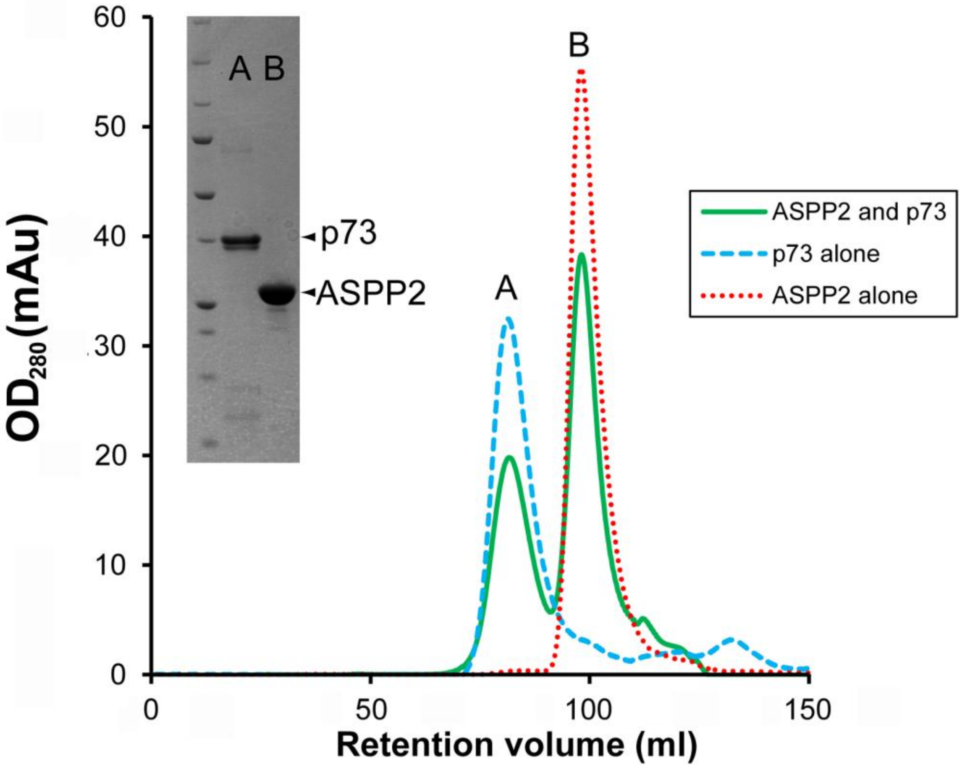

Figure S5. Dissociation of the p73-ASPP2 complex upon gel filtration. Different p73 constructs were mixed with ASPP2 (residues 891-1128) and analyzed by size exclusion chromatography in an attempt to purify a stable and homogeneous p73-ASPP2 complex for crystallization. However, co-elution of the two proteins was not observed. An example experiment using a HiLoad 16/60 Superdex 200 column (GE Healthcare) is shown (green line) in which 1.6 mg of tetrameric p73 (residues 112-400) was combined with 1.33 mg of ASPP2 (a 1:1 molar ratio) and run in gel filtration buffer **(**10 mM HEPES pH 7.5, 150 mM NaCl, 0.5 mM TCEP). The two proteins eluted as separate species (labeled A and B) as shown by SDS-PAGE. Dashed blue and red lines show the elution profiles of the individual p73 and ASPP2 proteins, respectively.

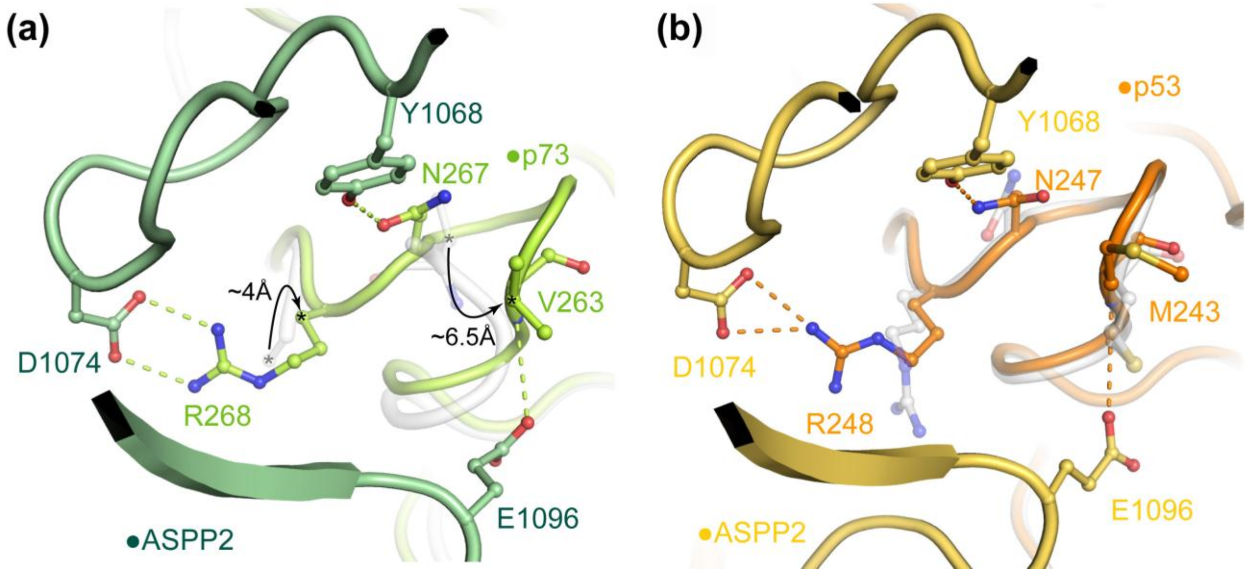

Figure S6. Structural changes in the L3 loop upon binding of ASPP2. (a) Part of the p73 L3 loop and the R268 side chain were disordered in the unbound p73 DBD structure (shown in white and semi-transparent). Superposition of the p73-ASPP2 complex (shown in light green / green) reveals that significant structural reorganization of the L3 loop occurred upon ASPP2 binding. Arrows indicate the movement of p73 residues V263 and R268 upon binding ASPP2. Dashed lines highlight the stabilizing interactions formed at these positions. **(b)** A similar comparison of the structures of monomeric p53 (white) and the p53-ASPP2 complex (light orange / orange) reveals no changes to the protein backbone, but a shift in the side chain rotamer of p53 R248.

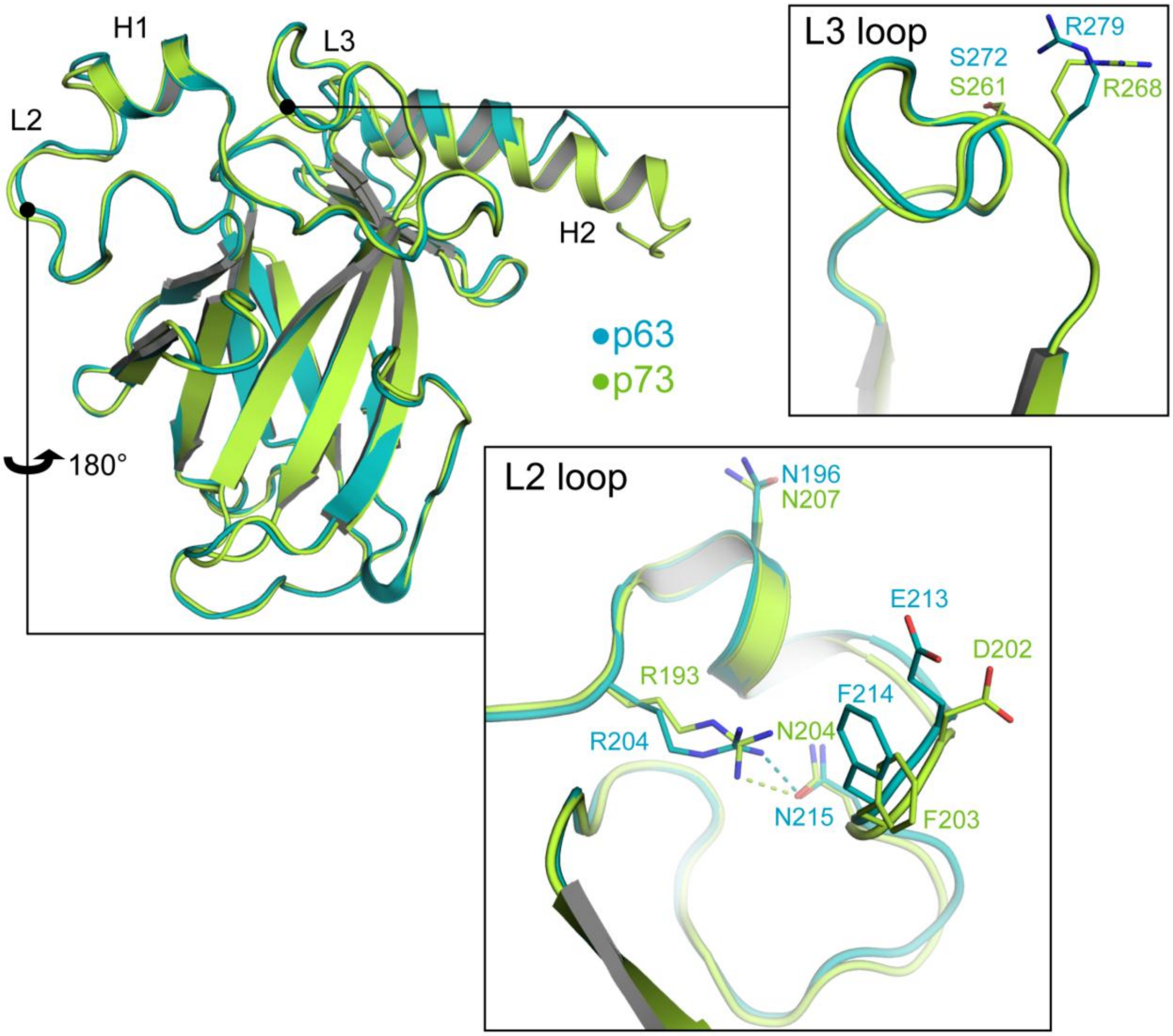

Figure S7. Conservation of the variant L2 loop in p63. Superposition of the DNA-binding domains of p73 (green, PDB 4A63) and p63 (cyan, PDB 3US0) reveals strong conservation of the overall fold as well as the L2 and L3 loops (boxed). The crystal structure of p63 was solved in complex with DNA and is matched more closely to the ASPP2-bound p73 domain (shown above, Cα RMSD 0.7 Å over 195 atoms) than the unbound p73 structure (Cα RMSD 1.04 Å over 192 atoms). The L2 loop in p63 also contains a two-residue insertion and adopts a similar pattern of hydrogen bonding to p73. The side chains of selected residues are shown as sticks. For clarity the L2 loop structure is rotated by 180° in the lower inset box. Note that p73 R193 and R268 are equivalent to p53 R175 and R248, respectively.
